# Collective intelligence in teams: Contextualizing collective intelligent behavior over time

**DOI:** 10.3389/fpsyg.2022.989572

**Published:** 2022-10-31

**Authors:** Margo Janssens, Nicoleta Meslec, Roger Th A. J. Leenders

**Affiliations:** ^1^Department of Organization Studies, University of Tilburg, Tilburg, Netherlands; ^2^Jheronimus Academy of Data Science, Tilburg University, ‘s-Hertogenbosch, Netherlands

**Keywords:** collective intelligence, team processes, interaction, team behavior, time, environment

## Abstract

Collective intelligence (CI) in organizational teams has been predominantly understood and explained in terms of the quality of the outcomes that the team produces. This manuscript aims to extend the understanding of CI in teams, by disentangling the core of actual collective intelligent team behavior that unfolds over time during a collaboration period. We posit that outcomes do support the presence of CI, but that collective intelligence itself resides in the interaction processes within the team. Teams behave collectively intelligent when the collective behaviors during the collaboration period are in line with the requirements of the (cognitive) tasks the team is assigned to and the (changing) environment. This perspective results in a challenging, but promising research agenda armed with new research questions that call for unraveling longitudinal fine-grained interactional processes over time. We conclude with exploring methodological considerations that assist researchers to align concept and methodology. In sum, this manuscript proposes a more direct, thorough, and nuanced understanding of collective intelligence in teams, by disentangling micro-level team behaviors over the course of a collaboration period. With this in mind, the field of CI will get a more fine-grained understanding of what really happens at what point in time: when teams behave more or less intelligently. Additionally, when we understand collectively intelligent processes in teams, we can organize targeted interventions to improve or maintain collective intelligence in teams.

## Introduction

Intelligence has captured the attention of scientists and practitioners because it portrays a desired state: we want to be called intelligent, show intelligent behaviors, and work in intelligent teams or organizations. Intelligence is an established concept at an individual level, but even there, various approaches and debates exist on how intelligence should be defined or operationalized (Deary, 2012; Funke, 2022). In general, researchers agree that individual intelligence is some sort of mental capability that involves the understanding of complex ideas, the reasoning about various courses of action, planning, and the solving of problems. Intelligence reflects a deeper capability of understanding the environment and making sense of what needs to be done (Funke, 2022). In fact, the etymology of the word *intelligence* highlights this very aspect: the Latin term “intelligentia” includes the verb “legere” (meaning: “to select, to choose”) and “intellegere” (meaning: to “understand, comprehend”) (Holm-Hadulla and Wendler, 2022). At its core, individual intelligence revolves around one’s ability to make sense of the world and circumstances and to actively select appropriate ways of dealing with challenges that require solutions.

Understanding individual intelligence has been very useful in understanding why some people thrive in our modern world, whereas other people struggle. In order to achieve a more complete understanding of this phenomenon, the concept of individual intelligence has been extended with multiple types of intelligence, beyond cognitive intelligence: e.g. emotional intelligence and social intelligence. One important impetus for some of these types of intelligences is the fact that much of human life occurs in social groups, not in isolation. In fact, in settings such as work teams — where team members work interdependently to achieve a common objective — individual intelligence is not always a strong predictor for important team outcomes. Teams are widely implemented in a variety of organizational settings because they can tap into a broad set of knowledge and capabilities to solve (complex) problems that are otherwise difficult to tackle by individuals (Glassop, 2002; Aguinis and Kraiger, 2009). However, this does not mean that a team is collectively highly intelligent. Although teams have at their disposal various bases of knowledge and member experience, the team is also highly dependent on the ability of its members to integrate these resources, combine individual knowledge into joint problem-solving solutions, and the joint ability to implement the solution in practice. Whereas much of the academic findings regarding collective intelligence are based on studies in laboratory settings (where groups are asked to solve, e.g., cognitive puzzles that can also be performed by individuals), real teams in organizations often need to find approaches to tackle complex, multi-faceted problems that do not have a single best answer. It requires both coordinated effort to come up with a feasible plan and to implement this plan over time. In essence, the ability for teams to act truly intelligently is embedded in the repertoire of possible between-member interaction patterns that a team has (or is able to develop over time). In essence, we argue that an important way to advance our understanding of collective intelligence is to focus on the behavioral side of teams.

To do this, we start this manuscript by reviewing two research streams that have largely shaped the collective intelligence literature. In one stream, CI is defined as the general ability of a group to perform a wide variety of cognitive tasks (Woolley et al., 2010; Engel et al., 2014; Kim et al., 2017; Mayo and Woolley, 2017), resulting in a *c-factor*. This c-factor is similar to defining and measuring individual intelligence in terms of the general intelligence cognitive testing (Spearman, 1904; Fletcher and Hattie, 2011). A second research stream focuses on *synergy* and proposes that CI arises when a team outperforms the aggregated capabilities of individual team members (Kurvers et al., 2015a,b). Teamwork is assumed to provide advantages compared to individuals working alone, resulting in process gains or “synergy” in teams (Larson, 2010; Hertel, 2011; Mojzisch and Schulz-Hardt, 2011; Volmer and Sonnentag, 2011). A consistent finding in both research streams is that teams vary considerably with respect to their collective intelligence levels. This indicates that there is potential for teams to achieve high levels of intelligence, nevertheless it is not yet fully clear why some teams behave more intelligent than others. A recent meta-analysis found that an important predictor of CI is the collaboration process between team members (Riedl et al., 2021), hinting at the vital relevance of interpersonal interaction for CI. It is exactly the between-member interaction processes that is the focus of our perspective in this paper.

In this paper, we start from the two established streams of CI and subsequently propose three main theoretical extensions. One extension relates to shifting the focus from outcomes (“teams that find the best solutions are the most collectively intelligent”), to a behavioral focus (“teams that solve problems in a mutually intelligent manner are collectively intelligent”). Next, we discuss giving ‘time’ a more central role in the CI conceptualization. Time plays a role both in the way the team interaction process unfolds and in how a team develops its collective intelligence. Finally, we suggest a stronger focus on the importance of the environment, because behavior can only be evaluated as intelligent if it matches (changing) environmental needs (Raab and Gigerenzer, 2005). Central in our argument is the idea of the team’s interaction process. The established CI streams suggest that the way in which team members interact is important to the team’s ability to be collectively intelligent, but they do not measure and operationalize the overall process explicitly. Rather, these studies focus on aggregated process measures as antecedents to predict (intelligent) team outcomes. To illustrate, a previous insight in the CI literature shows that equality of speaking time (aggregated over the full performance episode) predicts team performance (Woolley et al., 2010). However, such a summary index reduces the richness and complexity of the real life collaboration process, in which at some points in time, more equality speaking episodes take place, while at other times more centralized speaking episodes might be present. Therefore, we suggest in our process-oriented CI approach to disaggregate the intelligent process in relation to (changing) environmental demands and evaluate at each point in time how the team behaves as more or less intelligent.

Overall, this manuscript suggests a shift in focus when studying the complex phenomenon of CI by advocating a process-oriented perspective regarding actual team behavior relative to environmental demands. Given the above, we define CI in teams as an *unfolding process of collective behaviors (i.e., content, rhythm, participation), originating in coordinated inter-individual behavioral acts, in alignment with the environment in which the team operates and focused on the achievement of joint objectives*. We give theoretical primacy to *collective behavior*, which refers to any observable movements, interactions, and communications in which teams engage (Baumeister et al., 2007; Lehmann-Willenbrock and Allen, 2018). We argue that a team’s intelligence is more than a fixed concept, reflected in a static performance score. Rather, we propose a more temporal approach in which team intelligence emerges through unfolding communication, while the team aligns its behavior with the requirements of the environment.

Below, we will briefly sketch the two research streams that the current CI field is based on and suggest three extensions to the field, focusing on how CI actually occurs and is shaped in real world organizational teams. From there, we identify several intriguing research directions that unlock the temporal aspect of process-oriented collective intelligence. We conclude this manuscript by presenting a variety of methodological considerations involved in this ambitious approach.

## Collective intelligence: A brief review of two foundational streams

The current CI literature has largely been shaped by two streams of research: ‘*c-factor’* and ‘*synergy*’. Although there are more research approaches in the CI literature at large, these two streams of research have been selected because they (1) define collective intelligence at the team level (i.e., wisdom of the crowds is excluded from this review because of the higher level of analysis) and (2) explicitly define and measure CI (broader group process literature such as team learning and groupthink do not fit within the scope of our focused review). Below, we briefly establish the main approaches within these literature streams (c-factor and synergy). We do not aim to provide an all-encompassing overview of the literature in these streams; our objective is to establish their main tenets, to clarify how our suggestions build on and extend the status-quo in the field of CI.

### Collective intelligence as the c-factor

The c-factor research stream emanates from a seminal paper by Woolley et al. (2010). Similar to the general intelligence factor (“g-factor”) identified in individual intelligence testing (Spearman, 1904), this stream indicates the presence of a general ability factor for teams (“c-factor”) collectively performing a wide range of cognitive tasks (e.g., Mao and Woolley, 2016; Mayo and Woolley, 2017). The c-factor emerges from correlations among how well teams perform on a variety of cognitive tasks (Woolley et al., 2010). Additionally, the c-factor has been argued to predict future collective team performance on more complex tasks, which cannot be explained by the average individual intelligence of the team members (Woolley et al., 2010, 2015). One of the main predictors of the c-factor is ‘social perceptiveness’ or ‘social sensitivity’ of team members (Engel et al., 2014, 2015; Meslec et al., 2016), defined as the ability of team members to reason about the mental states of others (Baron-Cohen et al., 2001).

Although empirical support for the c-factor was found across a variety of studies (Engel et al., 2014, 2015; Kim et al., 2017; for a more comprehensive overview see Table 1), the c-factor also faced some controversy. In contrast to the original findings, Barlow and Dennis (2016), found empirical support for two dominant factors instead of a single ‘c-factor’. Further, Bates and Gupta (2017) could not replicate the original c-factor findings. Finally, Credé and Howardson (2017) showed statistical artifacts suggesting insufficient support for the existence of a c-factor construct after re-examining pooled data across six studies. Woolley et al. (2018) later countered the criticisms by pointing to misinterpretations in their scoring procedure and by pointing out that the assumptions underlying the simulation by Credé and Howardson (2017) did not match the majority of tasks that were actually performed.

**Table 1:** Representative sample of the collective intelligence factor research stream.

**Prior research**	**Definition collective intelligence**	**Research questions**	**Study number**	**Sample**	**Number of teams**	**Treatment**	**Research design**	**Major findings**
Woolley et al. (2010)	“the general ability of the group to perform a wide variety of tasks” p. 687	Does a collective intelligence factor exist for groups of people?	Study 1	General population United States	40	Face-to-face	Observational design using correlations	- Empirical support for existence c-factor - Individual intelligence score is not correlated with c-factor - C-factor predicts group performance better than average or maximum individual intelligence - Average social sensitivity predicts c-factor - Number of speaking turns is negatively correlated with the c-factor
			Study 2		152			
Engel et al. (2014)	“It is a measure of the general effectiveness of a group on a wide range of tasks” p. 3	Does the c-factor arise in online groups and what is the role of social sensitivity?	Study 1	General population United States	32	Face-to-face	Observational design using correlations	- Empirical support for existence c-factor - Reading the mind in the eyes test predicts the c-factor in face-to-face and online conditions - Total amount of communication positively correlates with the c-factor
					36	Online		
Engel et al. (2015)	“is a property of groups that emerges from the coordination and collaboration of members and predicts group performance on a wide range of task” p. 3769	Does the c-factor emerge across a variety of settings?	Study 1	General population United States	68	Face-to-face; text chat	Meta-analytic design using factor analytic approaches and correlations	- Empirical support for existence of the c-factor in different cultural settings, across communication media and group contexts - C-factor is correlated with performance on complex tasks
			Study 2	German student sample	25	Face-to-face; video; voice; text chat		
			Study 3	Japanese organizational context	116	Online		
Barlow and Dennis (2016)	“an ability of groups to perform consistently well across a variety of group-based tasks’’ p. 685	How does the c-factor manifest itself in computer mediated communication structures?	Study 1	Student sample in Midwestern university business school	86	Online	Correlational	- No empirical support for the existence of the c-factor in computer mediated context
Bates and Gupta (2017)	“strong general ability or group IQ factor” p. 46	What allows groups to behave intelligently?	Study 1	Student sample	26	Face-to-face	Correlational	- Empirical support for existence c-factor, but individual IQ accounted for the majority of group-IQ differences
			Study 2	General population India	40	Face-to-face	Correlational	
			Study 3	General population Scotland	40	Face-to-face	Correlational	
Kim et al. (2017)	“ability of the group to perform across a wide variety of tasks” p. 2	Does the c-factor translates into the world of teams in online video games?	Study 1	Gamers in North America	248	Online	Correlational	- The c-factor predicts a team’s future performance in League of Legend game - Social perceptiveness is a significant positive predictor of the c-factor

A recent meta-analysis including 22 studies and 1,356 group found evidence for a c-factor (Riedl et al., 2021). The sample included various populations from university students to military personnel, online gamers and workers, showing the existence of c-factor across a variety of settings. The meta-analysis also showed that the strongest predictor of the c-factor is by far the group collaboration process (Riedl et al., 2021). The group collaboration process was operationalized as the group’s ability to reach agreement between member’s skills and contributions to a task and also the group’s ability to coordinate their work in order to complete a task.

### Collective intelligence as synergy

The concepts ‘team synergy’ and ‘collective intelligence’ are often used interchangeably (Wolf et al., 2015; McHugh et al., 2016; Mann and Helbing, 2017). More specifically, scholars refer to teamwork which can provide advantages described as ‘process gains’ or ‘synergy’ in teams (Kurtzberg and Amabile, 2001; Hertel, 2011) (e.g., greater creativity and multiple perspectives) compared to people working alone. The idea behind team synergy is that teams can go beyond the performance level expected based on the (aggregated) capabilities of its individual members (Hertel, 2011). The synergy literature distinguishes between weak and strong synergy. Weak synergy refers to the ability of the team to perform better than the average of its team members (Larson, 2010; Hertel, 2011), while strong synergy refers to the ability of the team to perform better than its best performing individual (Larson, 2010; Carey and Laughlin, 2012). This stream of literature uses one particular research paradigm: comparing individual performance with team performance (Taylor et al., 1958; Sniezek, 1989; Volmer and Sonnentag, 2011; for a more comprehensive overview see Table 2). In essence, the main argument for CI in terms of synergy is that intelligence emerges when the team collectively outperforms the (best) performing team member(s).

**Table 2:** Representative sample of the strong and weak synergy literature stream in teams.

Prior research	Research questions	Sample	Number of teams	Treatment	Research design	Major findings
Carey and Laughlin (2012)	Are 3 person groups necessary and sufficient to perform better than the best individual on highly intellective tasks?	Students at University of Illinois	40404040	2 person team	Experimental study	The results suggest that groups of 3 members are necessary and sufficient to perform better than the best of an equivalent number of individuals on solving intellective problems. Empirical evidence for strong synergy
				3 person team4 person team5 person team		
Volmer and Sonnentag (2011)	Do experts in task and team functions predict team performance over and above the team’s average expertise level?	Software development teams from 28 different organizations in Germany	29	Expert vs team performance	Longitudinal, multi-source data	Experts positively predicted team performance 12 months later over and above team’s average expertise level No evidence for synergetic effects
	
	
	
Sniezek (1989)	What is the relationship among individual predictive judgement accuracy, confidence, influence and group judgment?	Students MBA	18	Individual judgement vs group judgement	Longitudinal within subject design	Group judgements are significantly more accurate than mean or median individual judgements Empirical evidence for strong synergy
Vollrath et al. (1989)	Do groups recall and recognize information better than individuals across a variety of measures and decision conditions?	Student sample university of Illinois	161	Individual or group decisionIndividual or group memory performanceDecision-then-memory or memory-then decision task sequence	2x2x2 between subject factorial design	Groups recalled and recognized information better than individuals across a variety of measures and decision conditions Empirical evidence for synergetic effects
Taylor et al. (1958)	What is the effectiveness of group brainstorming?	Yale University undergraduate students	12	Individual vs group condition	Controlled experimental study	Interacting groups generated significantly fewer ideas than pooled individual ideas No evidence for synergetic effects in brainstorming tasks



Within the synergy stream there are two main approaches. In the first approach, scholars pool *individual* responses by combining independent judgements of individuals (e.g., Wolf et al., 2015; Kurvers et al., 2015a). For example, Bettencourt (2009) describes the importance of having sufficient independence amongst judges to prevent people from copying reactions of others, and ensure they provide independent judgements. Similarly, Wolf et al. (2015) describe the need for independent assessment of multiple radiologists in a final decision for detecting breast cancers within patients. This approach assumes that team members do not interact while collaborating and consequently construct their contributions independently. Accordingly, Steiner (1972) concludes that some team tasks require simple pooled individual aggregations and are additive in nature. However, individual behavior does not always simply combine to determine the behavior of the team (Goldstone and Gureckis, 2009). Interaction is a key feature differentiating a team from an aggregate of individuals: one person’s behavior forms the basis for another’s response (Driskell and Salas, 1992). Likewise, McGrath (1984) states that the central feature, the essence of a team, lies in the *interaction* of its members. This is exactly what the second approach within the synergy stream emphasizes: teams outperform the individual (and hence are collectively intelligent) because of what happens in the team’s explicit communication (Larson, 2010). Previous research has focused on disentangling decision rules guiding the team’s interaction, ultimately fostering the team’s synergy. Decision rules are prescribed norms, guiding the interaction of team members and influencing how information is communicated and integrated (Meslec et al., 2014). For instance, the ‘collaborative decision rule’ encourages opinion sharing and equal participation of all group members during discussions (Curşeu et al., 2013). Another decision rule is the ‘majority rule’ reflecting a voting system in which the team adopts the decision made by the majority of members (Montes de Oca et al., 2011; Wolf et al., 2015). These examples demonstrate a first effort in disentangling how the team interacts to solve the tasks at hand in relation to its intelligence. Although most of the ‘CI as synergy’ literature states ‘intelligence’ lies in the quality of the outcome produced by the team (Baruah and Paulus, 2009; Hertel, 2011), some however emphasize that the decision rules themselves are intelligent (Wolf et al., 2015).

### The status-quo in the field and the behavioral approach

From the two main research streams that have largely defined the CI literature to date, we draw a few conclusions regarding the current state of the field. Both streams agree that collective intelligence is real, is important, and requires systematic investigation. Although researchers may differ in their approaches, they uniformly argue that teams can be intelligent - and that some teams achieve this better than other teams. Both streams consider this variation as an indicator that collective intelligence exists beyond anecdotal evidence. Additionally, accumulating evidence shows that the quality of interactions displayed by team members is key in explaining collective intelligence. For example, amount of communication, equal participation to group discussions, and group collaboration process have been found to be associated with the c-factor (Woolley et al., 2010; Engel et al., 2014; Riedl et al., 2021). Similarly, in the synergy stream, alterations of group interactions (e.g., through decision rules and norms) were associated with changing levels of synergy (Montes de Oca et al., 2011; Wolf et al., 2015).

We build our suggestions for a more behavioral view of collective intelligence from these joint findings, namely that collective intelligence is real, and it resides in the interactions between the team members. We base our arguments on collective intelligence in organizational teams, but they apply more broadly. Teams in organizations are often tasked with assignments that go beyond the ability of individual team members (e.g., Kratzer et al., 2004; Yu, 2005; Mathieu et al., 2017), because the task requires more time or more diverse knowledge and expertise than any individual in the organization has. The consequence of this is that team tasks in organizations necessarily require collaboration between the team members. Distinct from students jointly finding a solution to a solvable game or puzzle in a lab session, real organizational teams are often tasked with complex, multi-faceted, ambiguous tasks where the implemented solution has real implications for those involved (e.g., effect on sales, effect on the speed of product development).

We therefore conceive of collectively intelligent teams as those teams where members jointly identify and make sense of problems/issues/tasks that require solving, mutually coordinate activities, and jointly are able to implement their chosen solution. This view has several research implications, resulting in three main extensions that are outlined below.

## Extensions of the CI stream of research

### Extension 1: From intelligence-as-outcomes to intelligence-as-behavior

The lion’s share of the current CI literature defines and measures collective intelligence through the performance of a team; the central argument is that teams that consistently produce good outcomes, are collectively intelligent. Although we believe that higher collective intelligence will often lead to higher performance, we do not believe that outcomes reflect collective intelligence *per se*. Hence, we suggest that the field is better served by focusing on the interaction process that the team uses during their problem-solving activities, rather than mainly on the final outcomes.

A first argument focuses on the substantive nature of collective intelligence in teams. The essence of a team lies in the interactions between its members, and most real-life team tasks necessarily require the concerted efforts of team members with different backgrounds, expertise, and abilities. Thus, it becomes obvious that (much of) the collective intelligence of organizational teams is rooted in the ability of the team to organize, collaborate, and coordinate appropriately. Therefore, we argue that understanding exactly how teams differ in their internal organization (in terms of the patterns of interaction between the team members) will get researchers closer to the core of what really makes teams collectively intelligent. In sum, we argue for looking into what the team does at each point in time and evaluate its intelligence in terms of team behavior.

Another reason why moving away from outcomes advances CI research, is because performance scores tend to assume the existence of an ‘optimal solution’ or a ‘right answer,’ which does not capture the complexities of today’s team functioning. Real world teams operate in unpredictable and uncertain conditions that change over time (Stagl et al., 2006; Ramos-Villagrasa et al., 2018; Hoogeboom and Wilderom, 2020). That is, the team’s product or outcome may ultimately not be attained due to external or internal contingencies. A single ‘best answer’ occurs mostly in trivial, contrived settings, while the construct of CI has relevance in a broad number of organizational settings. Just as highly intelligent individuals do not always reach the “correct” solutions, we argue that team intelligence should be assessed by the way team members collaborate over time in their quest to find an appropriate (not necessarily best) solution, rather than by whether their solution is optimal. Apart from the question of whether optimal solutions are relevant in business settings (Simon and Barnard, 1947; Brown, 2004), we contend that collectively intelligent teams will have a higher probability than teams lacking collective intelligence to develop feasible and appropriate solutions to complex problems, and will be more likely to do so repeatedly over time.

A final argument in favor of this shift is methodological. We agree with authors who argue that constructs should be defined and understood independent of their effects (Antonakis et al., 2016; Alvesson, 2020). Studying the underlying nature of a phenomenon while measuring through the phenomenon’s outcome has shortcomings. Mathematically, this approach bears the dangers of confusing a construct with its mediators, moderators, confounding variables, and spuriously correlating variables. This risk diminishes as the same patterns are found across an increasing set of studies. However, equating a concept with its consequence will still be of little help to understanding the antecedents and nuances of a concept.

### Extension 2: From static to dynamic evaluations of collectively intelligent behavior

Our second extension reflects a conceptual shift towards a focus on dynamic aspects of collective intelligence. By its very nature, CI takes time in order to develop and solidify and thus needs to be understood in a temporal manner (Ballard et al., 2008; Gorman et al., 2017). Team members need to make sense of the complex task at hand, share and discuss information and ideas and co-construct knowledge, develop alternative strategies to find appropriate solutions, coordinate and integrate to actually develop feasible solutions, weigh alternative solutions against each other, reach a shared decision on one (or more) strategy solutions, and, where applicable, implement the chosen solution(s). In sum, CI tends not to emerge in a single moment, but rather through a series of interactions unfolding over time (Allen and O’Neill, 2015) - possibly quite long stretches of time for organizational teams. Unfortunately, the vast majority of CI research builds on static glimpses of team performance that occur at a single point in time, assuming that various levels of intelligence are due to collective behaviors, without actually measuring them. Conclusions drawn from these investigations do not shed light on the dynamic, unfolding nature of the collective intelligent team process that may distinguish intelligent teams from less intelligent ones.

Our suggestion is in line with the multilevel theory of emergence, that encompasses a dynamic process of lower level units (team members) over time, coalescing to create a collective entity (intelligent behavior) at a higher level of analysis (Kozlowski and Klein, 2000; Waller et al., 2016; Carter et al., 2018). Emergence theory emphasizes the processes embedded in dynamic interactions amongst units (i.e., the interactions between the team members) and stresses that it takes time to develop an entity (i.e., intelligent behavior) at the higher collective level (Kozlowski et al., 2013). Hence, we argue that CI needs to be conceptualized as multilevel and dynamic, focusing on how intelligent team behavior emerges over time across levels of analysis.

### Extension 3: Acknowledging the role of the environment

As explained in our overview of the CI literature, the c-factor approach is based on the idea of a single ‘collective intelligence factor’ across settings. The argument for this approach is that teams with a high c-factor are expected to perform well across a wide range of tasks, regardless of the task or conditions they will encounter in the future. Instead, we suggest that the CI literature should develop a focus on the relationship between teams and their environments. For instance, how CI unfolds in surgical teams differs substantially from how it unfolds in a sales unit team. In particular, the interpersonal behaviors that are required of a surgical team to solve medical tasks during routine surgery will largely be based on protocol, routine, and standardization. However, when a patient goes into unexpected cardiac arrest, or unexpectedly and prematurely wakes up from anesthesia, the team’s interpersonal behaviors will require some level of improvisation, more speed, and impromptu problem-solving (Gorman et al., 2012). During unexpected crisis situations, flexible, non-standardized communicative patterns that reorganize routines is often an intelligent approach to break out of normal structures and improvise (Stachowski et al., 2009; Bechky and Okhuysen, 2011). Therefore, different conditions require different interaction processes for the team to intelligently solve the issues at hand. Collective intelligent behavior is contingent on its environment, as certain team behaviors may not be viable given a particular task or situation (Kämmer et al., 2014). Thus, collective behavior can only be judged as intelligent if we evaluate that behavior against a broader set of environmental needs in which the collaboration takes place.

Incorporating the environment in the conceptualization of a team’s intelligence aligns with the theory of ecological rationality (Goldstein and Gigerenzer, 2002). Ecological rationality investigates which behaviors are better than others in a given setting; ‘better – not best – because in large worlds optimal behaviors are unknown’ (Gigerenzer and Gaissmaier, 2011, p. 456). Collective behavior is ecologically rational to the degree that it is adapted to the structure of the environment (Gigerenzer and Todd, 1999). Subsequently, specific team interactions are not good or bad *per se*, rather they are more or less appropriate to the environmental conditions in which that behavior takes place (Gigerenzer, 2004). No single behavior works at all times, just as a hammer does not work for all home repairs (Gigerenzer, 2015).

**Figure 1:**
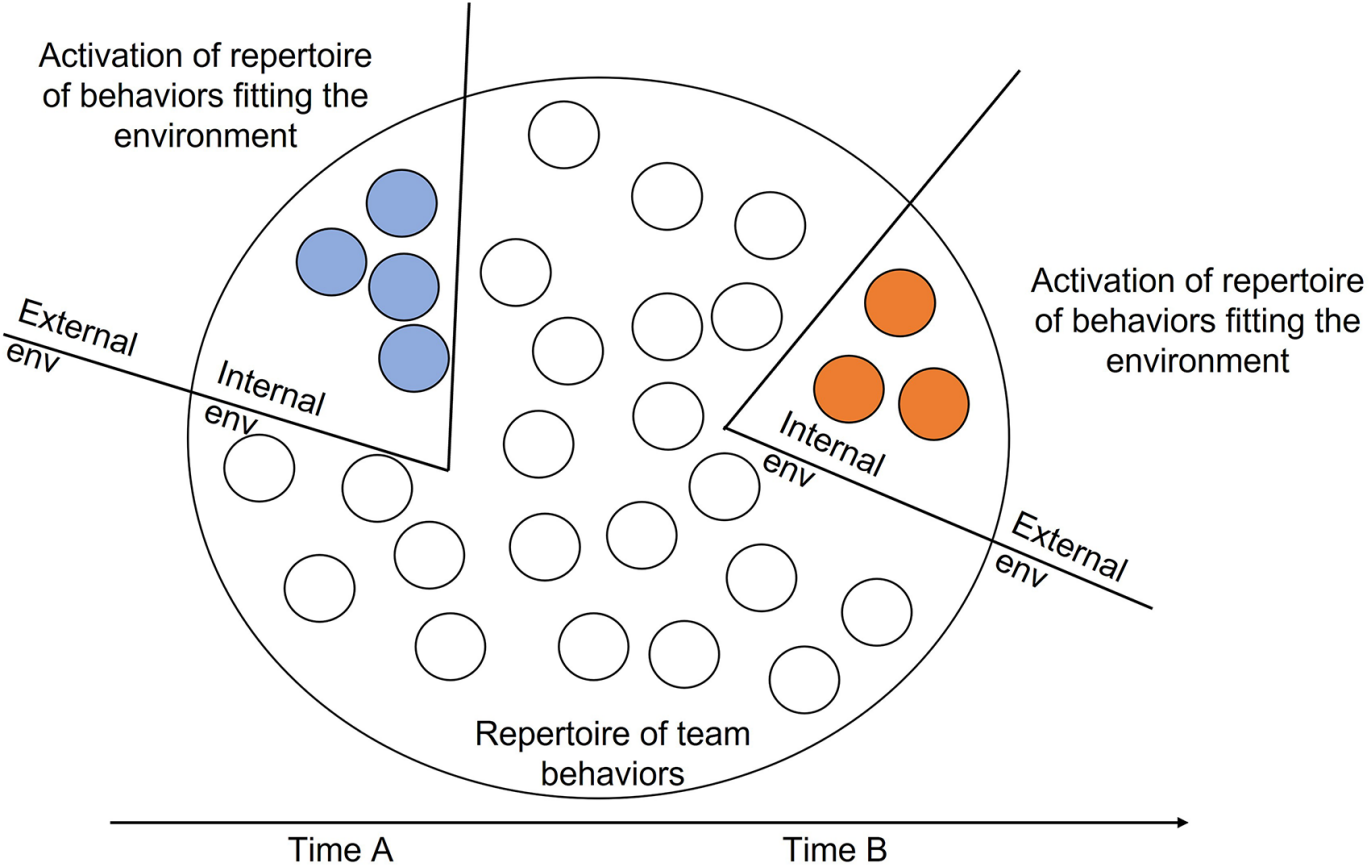
How a team’s behavioral repertoire aligns with its environment.

As we contend, intelligent teams engage in (adaptive) collective behaviors by matching their interaction patterns to fit the nature of the environment (Waller, 1999; Lei et al., 2016), or - where feasible - actively shape the environment to develop a match with the collective behavior (Ancona, 1990; Marks et al., 2005). We note that we conceptualize “environment” broadly and consider both internal and external environmental demands: the team needs to deal with ‘challenges’ of what happens either outside or inside the team boundary (Maloney et al., 2016; Johns, 2018). Teams must constantly update their repertoire of collective behaviors in relation to their environment (for a visual representation see Figure 1).

*External needs* are located in the environment outside of the team’s boundary, usually at a higher level of analysis (Mowday and Sutton, 1993; Maloney et al., 2016). Although teams usually have only limited control over external conditions, these are important given their role in guiding collective behavior. In concert with the framework of Mowday and Sutton (1993), we distinguish between *proximal* external needs that are situated closer to the team (e.g., organizational culture) and *distal* external needs (e.g., industry). An example of proximal external needs includes the strategy and core values of the organization. When the core values of the organization focus on creativity and innovation, collective behaviors in teams that enhance creative thinking (e.g., low centralization in interaction) would be an intelligent behavioral pattern to follow (Leenders et al., 2007a,b; Kratzer et al., 2008), whereas collaboration patterns aimed at maintaining routines and efficiency are less likely to stir team-level creativity and are a sign of a team that is behaving in a much less collectively intelligent manner within this organizational environment.

*Internal needs* are situated within the team boundary, originating from within the team itself (Maloney et al., 2016; Georganta et al., 2019). For example, a change in team composition regarding the *loss of a team member* requires the team to collectively respond (e.g., redistributing roles and workload) (Siegel Christian et al., 2014). Another example of an internal need is when a software development team faces a critical software failure during a development project. In this example the team must temporarily refocus on finding solutions to the error, before it can continue with the project execution. Collective intelligent behavior in this case is the team’s ability to recognize the changing needs, to shift focus to the new/unexpected specific task and restructure its internal collaboration process in order to tackle the software error (for example by organizing a collectively divided search for causes of the error in the code).

The number and heterogeneity of components in the environment that teams must engage with and understand, in addition to managing (conflicting) relationships amongst these components, are the foundations of grounding teams’ collective responses. Collective intelligent teams navigate this environmental complexity by actively and appropriately scanning their internal and external environment and consequently behaving collectively so their actions and interactions fit the variation in the environment (Wiersema and Bantel, 1993). In some cases, the interpersonal dynamics and member characteristics (internal demands) mainly drive the collective behavior, while in other cases the competitiveness of the industry is one of the main drivers for collective behavior (external demands). The challenge is to consider at which level(s) environmental demands are most likely to matter for the team collaboration. Environmental demands might change over time, yet collective intelligent teams are able to align their internal process with such changes.

The team environment not only shapes which collective behavior is more or less intelligent, but teams can often shape their environment as well. Although most studies consider environmental demands as requiring modified collective behavior from the team (e.g., Gersick, 1988; Waller, 1999; Lei et al., 2016), some studies consider how the team reaches out to its environment to potentially modify external and/or internal needs (Ancona, 1990; Marks et al., 2005). Teams can reduce uncertainty by negotiating malleable environmental conditions, for example proactively increasing resources by lobbying for additional human capital to manage the team’s workload. We believe that collectively intelligent teams not only respond smartly to their (internal and external) environment, but also actively try to manage the environment to support the suitability of the team’s internal processes.

## A behavioral perspective on collective intelligence

The objective of this paper is to outline a different approach to the understanding of collective intelligence in teams. Our approach shifts the focus from outcomes-as-CI to process-as-CI. Although teams that embody collectively intelligent interaction processes are more likely to consistently deliver high quality output, we argue that the collective intelligence of teams is reflected by the intelligence of their internal processes, not by their output. Just like the intelligence of an individual enables him/her to perform well at an IQ test, the person’s (percentage of) correct answers given at the test is not that person’s intelligence, they are only the consequence of it. We suggest adopting a similar approach to the study of team intelligence: the intelligence of the team is the ability to consistently act “smart” *as a team*, it is not the output, or number of correct answers given by a team in a test. Thus, the more appropriately team members interact with each other (i.e., who does what with whom and when), building interaction routines, making team processes sufficiently efficient while retaining the cognitive and procedural flexibility to adapt to changing environmental demands, the more we view this team as being collectively intelligent. This is why we define collective intelligence as an *unfolding process of collective behaviors (*i.e.*, content, rhythm, participation), originating in coordinated inter-individual behavioral acts, in alignment with the environment in which the team operates and focused on the achievement of joint objectives*.

The question remains which of the interpersonal team members’ behaviors sufficiently describe the elements of collective intelligent behavior in teams. We suggest that collective behaviors unfold mainly through team interaction, defined as any verbalization and nonverbal action intended for collective action and coordination (Zellmer-Bruhn et al., 2004). Communication is the primary mechanism for interaction, serving as a conduit through which information gets exchanged (Marks et al., 2000) and is of particular significance for the teams’ intelligence because ‘it is the vehicle through which the majority of collaboration is accomplished’ (McComb and Kennedy, 2020, p.2). Building on the framework of McComb and Kennedy (2020), the communication processes in terms of collective behavior can be divided into three components: the *content* of the topics discussed (e.g., planning how to approach the task), the *degree of participation* (e.g., equality of speaking time), and *the rhythm* of communication (e.g., pace, speed).

The *content* of the interaction focuses on the ‘what’ of the conversation. The subject of what is being discussed is often important for determining whether the team engages in collective intelligent behavior. For example, interaction content can be oriented towards developing a common representation of the problem, generating possible solutions (DeChurch and Mesmer-Magnus, 2010), or structuring and organizing the discussion. These content behaviors can be more or less intelligent given their timing: developing a common representation of the problem at hand is generally more suitable or ‘intelligent’ at the beginning of a collaboration period than at the end. The *degree of participation* reflects the ‘who’ in terms of the actors involved. Conversations can be concentrated amongst only a few team members, or equally distributed among all team members (Warner et al., 2012). At the same time, teams can benefit from equality in participation during some periods of the execution of the team task, in combination with episodes of concentrated centralized ‘speak-ups’ during other periods (e.g., in multidisciplinary decision-making teams, when experts in the field need to speak up regarding particular topics). Lastly, between-member interaction is characterized by its rhythm or pace and intensity. During crisis situations, high pace and intense burst of interaction can be highly intelligent (combining important pieces of information rapidly) while in stable situations, such as reflective meetings, a slower pace may be more appropriate. In sum, we expect that collective intelligent teams are aware of these three communication aspects and adjust them in such ways that the team members’ processes correspond to the needs of the environment at that time.

At this point, it might be insightful to provide a practical example of how our suggestions extend and differ from the c-factor and synergy literature streams. We do so by putting forward a case of an actual organizational team we studied, showing how collective intelligence would be defined and operationalized in each research stream. Our example focuses on a multidisciplinary health care setting, in which a group of physicians come together on a weekly basis to discuss and decide on treatment plans for patients (see Table 3 for a comparison across research streams).

**Table 3:** Comparison of CI applications across c-factor, synergy as well as process-oriented view.

	C-Factor	Synergy	Process-Oriented CI
Definition	CI is defined as the general ability of the team to perform well across a variety of cognitive tasks. In the context of multidisciplinary health care teams, the underlying premise is that if the team collectively scores high on a generic collective ability test, it is collectively intelligent and hence can ‘transfer’ it’s intelligence to other contexts as well.	CI is defined as whether the team outperforms the (best) individual team member. In the context of multidisciplinary health care teams, a team would be collectively intelligent if it jointly makes better decisions regarding treatment plans for patients compared to when one physician comes up with a treatment plan individually.	Intelligence is defined as an unfolding process of collective behaviors (content, rhythm, participation) that originate in individual level behavioral acts, that are appropriate for the tasks that are assigned to the team and in alignment with the environmental needs in which the team operates. In the context of multidisciplinary health care teams, the team would be intelligent if the content of the conversation, the way in which they discuss, as well as who participates is appropriate and effective to solve the task the team is working on and is also in line with changing environmental needs. One important change in the environment in multidisciplinary health care teams is that patient cases vary in terms of complexity. In low complexity patient case discussions, a more fast-paced, standardized process with fewer people contributing to the discussion is often considered as an intelligent way of organizing (by medical experts). However, in complex patient case discussions, a low pace, with input from varied medical experts, combined with actively questioning one another is generally considered as intelligent behavior. In sum, a relevant/salient changing environmental need (i.e., complexity/rareness of disease) requires different ways of organizing interactional structure and thus the team needs to be adaptive towards changing environmental needs. Collective intelligence is now considered high for medical teams that easily shift between discussion formats as they move from case to case, whereas less intelligent medical teams would be more stuck to a single way of discussing, regardless of the complexity of each specific case.
Operationalization	Collective intelligence is measured by giving teams various cognitive tasks (e.g., spatial reasoning, mathematical, linguistics tasks). Factor analytic approaches are used to identify one latent underlying ability factor reflecting the team’s intelligence. The higher the performance across cognitive tasks, the higher the team’s intelligence and hence the better the team is expected to come up with suitable treatment plans, now and in the future. One assumption is that the performance of the team can be measured correctly and objectively.	Each physician would be asked to come up with a treatment plan for patients individually. Subsequently, the team would be asked to collectively come up with a treatment plan, following group interaction. The health care team would be evaluated as intelligent if the team comes up with a ‘better’ or ‘more suitable’ treatment plan for the patient, compared to the physician making the best decision individually. One underlying assumption is that individuals can do the task, so a team is not necessary. Also, it is assumed that it is possible to objectively judge which treatment plan is “best.”	In the process-oriented CI approach the researcher analyzes transcripts of who says/does what at what point in time during the medical team meeting and investigates how medical expertise is shared across patient cases. First, the evaluation must be made whether the content is aligned with the needs of the patient and whether sufficient information and relevant medical expertise is communicated within the team. Second, it is evaluated whether the experts speaking up are also the ones that would be expected to contribute given the background/complexity of the patient case. Lastly, the researcher would look at the rhythm or pace of the decision-making process; do team members follow logical sequences of decision making? Or is the conversation totally scattered? Is the conversational pace efficient and clear for members to follow? Each of these features must be evaluated in context to reflect on the intelligence of the behaviors that take place within the team. Having interpreted the appropriateness of the interaction process for each patient case, the researcher then assesses to what extent the team was able to adopt fitting discussion procedures over the course of the entire meeting (so across all patient cases/tasks that the team had to formulate a solution for). The better the team adjusted its discussion format to the requirements of the specific case, the more intelligent it was. Then, if it is possible to collect such data over multiple meetings, it can be assessed whether collective intelligent teams are indeed able to display the required procedural flexibility in later meetings as well.

Our proposed behavioral understanding of collective intelligence creates the opportunity for new research directions and methodological developments. Below, we will first present a series of research questions that can be addressed by prioritizing the team’s interpersonal processes. After that, we discuss methodological challenges and opportunities that arise when taking this research perspective.

## The road ahead: Future research directions

Taking a behavioral approach to collective intelligence shifts the focus to research questions that may differ from those currently addressed in the CI literature. There is surprisingly little known about which micro-level interaction processes support which problem-solving tasks, so this research question is both important and still largely unexplored. In general, it makes sense to expect that the specific elements of the team’s internal interaction processes will likely depend on the task at hand and on environmental demands. The main research question we address here is *how the interpersonal team members’ behaviors, that embody collective intelligence, vary across environmental conditions*. Related to this we wonder *which set of conditions might be coped with by similar sets of team behaviors whereas other conditions might require very different joint behaviors*.

There are many conditions that can affect which interpersonal processes are appropriate in a specific situation. These include team composition: a highly diverse team in terms of expertise and experience may benefit from different interaction patterns than homogenous teams. Another condition is team size: larger groups will more naturally split apart into smaller subgroups, hence an attempt to constantly mutually discuss and coordinate is often less desirable in large teams than in small teams. Team longevity may play a role too since teams where members have worked together for a long time can more easily build efficient routines, but are also more at risk of “forgetting” to challenge each other and will have a harder time integrating newcomers into their interpersonal routines (Katz, 1982; Esser, 1998).

Another condition that may be highly important is the extent to which environmental conditions are stable or unstable. The more stable the environment, the more the team can develop efficient routines and procedures. This is a sign of collective intelligence, as it shows that the team understands that the environment is unlikely to change, providing the opportunity to optimize internal processes. Routinizing interactions also allows teams to easily deal with changes in team member composition: the clearer the norms and procedures around who does what with whom and when, the more clarity there will be for newcomers regarding what is expected of them. Alternatively, the more unstable the environment, the more such routines and fixed expectations hinder the team in adapting to new environmental requirements. In these conditions, collective intelligent teams aim to create interpersonal procedural flexibility, which requires different ways of interacting (Kratzer et al., 2010; Schönrok, 2010).

In order to understand interpersonal processes in teams, there is a need to focus on the flow of interactions between the team’s members, considering (shifts in) e.g. pace, rhythm and order, rather than aggregating the actual process away by only considering averages and general summaries of a process that is dynamic at its core. The main research question here is *which temporal aspects of team member interaction and which resolutions need to be considered in operationalizing collective intelligence.* When teams need to solve tasks that require days, weeks, or longer to solve, there may be short term flows in the interaction (following a daily rhythm), but there will often also be an overarching dynamic over the course of the project. Intelligent teams will probably try to plan and schedule ahead and decide early on about the order and timing of various subtasks, while leaving enough slack in the schedule to account for unforeseen circumstances. In product development teams it is often the case that the team aims to be as creative as possible in the early stages (in order to generate as many feasible solutions as possible), and then, after one promising solution has been selected, aims to be as lean as possible in the later stages when the focus is on implementation. In other words, the intelligent way of organizing in the early stages revolves around stimulating effectiveness, whereas the later stages require interpersonal interaction aimed at efficiency. The collective intelligent interaction underlying these two rough phases are quite different and require shifts in their interaction process.

A second way in which time plays a role is in the questions *how does CI develop over time* and *to what extent is CI stable*. Teams learn which behaviors work best given a situation, based on prior collective experiences (Edmondson, 1999; Raes et al., 2015). Through this process of team learning, we posit that collective intelligent behavior may develop (non-linearly) over time. New teams may take some time for the members to get to know each other and to learn how to relate to one another vis-à-vis a specific joint task. It is likely that CI may then develop fairly quick, up to a point. From there, CI may plateau before it (gradually) increases. With changing environmental conditions, some teams may suffer a loss in CI and need to increase their interpersonal behavioral repertoire to cope with a wide range of conditions. This issue may be of particular interest to organizational practitioners wanting to understand *how teams maintain their CI.*

## Methodological challenges

Although we believe that an increased focus on interpersonal team member behavior can advance understanding of collective intelligence in teams, it is not necessarily straightforward how to incorporate the full agenda in empirical research. Focusing on actual behavior rather than on outcomes, requires the collection and analysis of fine-grained data. This poses several challenges and opportunities for methodological innovations. Below, we will briefly touch upon three main areas: collection, coding, and analysis of data.

### Data collection: Capturing high-resolution, longitudinal team interaction

In order to adequately map activity in team behaviors over the timeframe of a task, high sampling frequencies are needed (Klonek et al., 2016; Lehmann-Willenbrock and Allen, 2018). If we were to measure interaction only once or twice during the collaboration period, we would not be able to answer research questions such as how CI develops over time. Accordingly, studying CI in teams will benefit from unobtrusively capturing ongoing longitudinal interactions in real time - which translates to high-resolution datasets (Kozlowski, 2015; Klonek et al., 2016). Particularly, it is valuable to capture the trajectory of what has been said by one team member to one or more different team members at each point in time, to get a near continuous movie-like representation of the collaboration process (Leenders et al., 2016; Meijerink-Bosman et al., 2022a). Time-based sampling of interaction behaviors allows for in-depth analysis of what happens over time and when teams act more or less intelligently. We acknowledge that it requires effort to disentangle micro-level behavioral dynamics underlying the collaboration processes, especially in projects with longer time spans (e.g., months or even years). In this case, not only is infeasible to capture the full interaction details of what happens minute by minute, but it may also not be necessary. For teams whose tasks take long periods of time, measures of the interaction process may be gained by simpler means such as looking at minutes of team meetings to distill who met with whom, when, what was discussed and what was decided. Also, regular brief surveys or intermittent observations may be effective approaches. Other data collection tools that are frequently used to get a fitting image of the interaction dynamics inside the team include capturing electronic traces of team member interactions. Examples include email records (who sends a message to whom when), electronic badges (capturing co-location in rooms), company discussion boards (such as yammer), or message exchanges on project-specific software platforms.

Over time, as we perform more empirical studies on collective intelligence and develop a better overview of which aspects of team member interaction process are critical for collective intelligence, better data collection strategies can be designed and demarcated as well.

### Data coding: Behavioral coding schemes

Once we have collected data capturing who does what, with whom, when (at the resolution that fits with the team and the task at hand), we still do not have a dataset that allows us to analyze collective intelligent behaviors. First, we need to identify the actual behaviors taking place during the collaboration trajectory, characterize them, and evaluate them against relevant environmental demands (Lehmann-Willenbrock and Allen, 2018). In short, the interactional data needs to be coded to be able to subsequently make sense of the behavior.

In the literature, a variety of theory-based, validated coding schemes for measuring the fine-grained team interaction exist, distinguishing between mutually exclusive and exhaustive behavioral categories. The work of Robert Bales has been particularly important for the development of useful behavioral coding approaches (Bales, 1950; Bales and Strodtbeck, 1951; Bales et al., 1951; see Brauner et al., 2018 for an overview of team interaction coding schemes). Behavioral coding means that researchers or trained coders assign codes to behavioral acts using a predefined coding scheme (Klonek et al., 2020), resulting in an overview of the entire flow of conversational events exchanged among group members. This facilitates comparison within and across teams. A variety of software programs to facilitate the transcribing and coding has become available, such as MAXQDA (Kuckartz and Rädiker, 2019) and ATLAS.ti (Paulus and Lester, 2016). More recently, researchers are working on developing innovative deep learning algorithms to automatically code behavior in videos, which is promising for coding large amounts of data on interpersonal behavior in teams (Gibson et al., 2022). Time-stamped and behavioral coded data allows researchers to investigate how different team behaviors are interrelated and dependent on environmental demands, which is exactly what is needed to unravel the nature of CI.

### Analysis of interactional data

Having coded fine-grained longitudinal interactions, the focus in research projects can turn to the actual analysis of this data. Despite collecting high-resolution data, researchers too often aggregate fine-grained process data over time to form static summarized variables (Klonek et al., 2016). For instance, a previous insight in the CI literature shows that equality of speaking time (aggregated over the full performance episode) predicts team performance (Woolley et al., 2010). Such a summary index reduces the richness and complexity of the data to support ease of statistical analysis. However, this comes at the expense of precluding the researcher from truly capturing the effect of temporal dynamics (Klonek et al., 2016). Collapsed temporal data often oversimplifies reality, as the equality of speaking time is almost never constant over time. When variance across time is collapsed into a static summary indicator, this removes the potential to uncover temporal effects (Leenders et al., 2016).

Several researchers developed tutorials on how to analyze this complex type of interactional data (Dabbs and Ruback, 1987; Lehmann-Willenbrock and Allen, 2018; Nyein et al., 2020). Lag sequential analysis, pattern analysis, sequential synchronization analysis, and statistical discourse analysis have recently gained ground among team researchers and psychologists, in their efforts to achieve a good grip of the actual flow of interactions in organizational teams.

We briefly highlight a few other recent developments for the analysis of high-resolution time-stamped interaction data. First, we suggest relational event models as uniquely suitable as they have been developed to analyze time-stamped (or ordered, without the precise time-stamp) interaction patterns across members of a team (Quintane et al., 2014; Leenders et al., 2016; Pilny et al., 2016; Schecter et al., 2018; Mulder and Leenders, 2019; Meijerink-Bosman et al., 2022b). Relational event models are built on a simple idea: the rate at which two individuals interact at a specific point in time is determined by past team interactions. The statistical model itself is a simple event history model, but one that considers that observations are not independent of each other (because the intensity of the interactions may be affected by prior interaction). The result of this type of model is a set of variables that predict who interacts with whom, at what point in time (or in what order). These variables can then be taken as representative of the dynamic interaction patterns in the team. Subsequently, it can be assessed how appropriate these interaction behaviors are for the task and given the broader environment.

A machine-learning modeling approach known as THEME, which is quite different from the relational event model, was developed by Magnusson (1996, 2000). THEME detects specific patterns of event sequences (called “T-patterns”) which has been used in the study of organizational teams (Ballard et al., 2008; Stachowski et al., 2009; Zijlstra et al., 2012). The THEME approach searches for so-called “hidden patterns” emerging from the data, and that occur more frequently than would be expected by chance encounters —typically, a few dozen such patterns will be found in an analysis.

Finally, once we understand the behaviors of team members and the fine-grained manner in which they co-construct knowledge and information, we can move towards the use of innovative simulation techniques such as agent-based modeling (ABM) (Gómez-Cruz et al., 2017). Agent-based models are computational models in which agents as autonomous individuals behave in a given environment or space according to established rules (Bonabeau, 2002; van Veen et al., 2020). These models are simplified representations of reality defined by the researcher. To start with, the agents in the model are team members and can be defined with unique individual characteristics. Second, the agents interact with one another following specific predefined rules. Researchers can define the possibilities for each team member’s behavior, based on insights gained from the transcripts and coding of prior team collaborations, or take the output of any of the previously mentioned statistical approach as input for the ABM. The environment for each team member in the model is a simulated multidimensional space that can represent any physical, economic, or psychological features (Secchi, 2015). Subsequently, team members in the simulated space can act in a variety of ways given their characteristics - again, these rules are typically informed by the results from the previous statistical analyses. These rules are set to ‘program’ the team members so that they behave accordingly, given specific conditions (Secchi, 2015). By keeping the behaviors the same but, simultaneously, varying conditions (such as team composition, changing tasks, or adding or removing team members) it can be assessed to what extent specific interaction behaviors that are intelligent in one condition are equally intelligent under different conditions.

## Conclusion

Some teams are more collectively intelligent than others, but we are far from understanding the exact group processes or behaviors that might explain these differences. In this paper, we embrace a behavioral approach to CI that suggests to focus research on the dynamic interpersonal interactions between team members. This is where collective intelligence resides, hence we suggest that this is where we should focus our research attention on. These interactions can vary in terms of content (e.g., engaging in planning activities), participation (e.g., who is talking), and rhythmic characteristics (e.g., conversational pace). The behavioral repertoire employed by the team must be appropriate for environmental needs: either collective behaviors must be adapted such that they align with environmental needs, or the environment should be shaped such that the collective behaviors are better suited.

Besides presenting a plea to shift the focus of the field to a behavioral view, we also outlined that this approach opens up a series of new research questions and methodological challenges and opportunities. The collective intelligence field is closely connected to several other fields, such as organizational and group learning. In this vein, we strongly believe that taking this next step in the collective intelligence literature might also inspire adjacent fields to take a more behavioral approach.

## Author contributions

MJ, NM, and RL contributed to the conception and ideas of the paper. MJ primarily wrote the manuscript. All authors contributed to the manuscript revision, read, and approved the submitted version.

## Conflict of interest

The authors declare that the research was conducted in the absence of any commercial or financial relationships that could be construed as a potential conflict of interest.

## Publisher’s note

All claims expressed in this article are solely those of the authors and do not necessarily represent those of their affiliated organizations, or those of the publisher, the editors and the reviewers. Any product that may be evaluated in this article, or claim that may be made by its manufacturer, is not guaranteed or endorsed by the publisher.
